# Combination therapy of curcumin and alendronate modulates bone turnover markers and enhances bone mineral density in postmenopausal women with osteoporosis

**DOI:** 10.20945/2359-3997000000060

**Published:** 2018-08-01

**Authors:** Fatemeh Khanizadeh, Asghar Rahmani, Khairollah Asadollahi, Mohammad Reza Hafezi Ahmadi

**Affiliations:** 1 Shahid Beheshti University of Medical Sciences Shahid Beheshti University of Medical Science Tehran Iran Obstetrician/Gynecology, Shahid Beheshti University of Medical Science, Tehran, Iran; 2 Ilam University of Medical Science Ilam Iran Ilam University of Medical Science, Ilam, Iran; 3 Ilam University of Medical Sciences Faculty of Medicine Departament of Social Medicine Ilam Iran Clinical epidemiology, Departament of Social Medicine, Faculty of Medicine, Ilam University of Medical Sciences, Ilam, Iran; 4 Ilam University of Medical Science Department of Pathology Ilam Iran Department of Pathology, Ilam University of Medical Science, Ilam, Iran

**Keywords:** Post menopause, osteoporosis, alendronate, curcumin, bone mineral density

## Abstract

**Objective:**

This study evaluated the effects of combination therapy of curcumin and alendronate on BMD and bone turnover markers in postmenopausal women with osteoporosis.

**Subjects and methods:**

In a randomized, double-blind trial study, 60 postmenopausal women were divided into three groups: control, alendronate, and alendronate + curcumin. Each group included 20 patients. Total body, total hip, lumbar spine and femoral neck BMDs were measured by dual-energy X-ray absorptiometry (DXA) at baseline and after 12 months of therapy. Bone turnover markers such as bone-specific alkaline phosphatase (BALP), osteocalcin and C-terminal cross-linking telopeptide of type I collagen (CTx) were measured at the outset and 6 months later.

**Results:**

Patients in the control group suffered a significant decrease in BMD and increased bone turnover markers at the end of study. The group treated with only alendronate showed significantly decreased levels of BALP and CTx and increased levels of osteocalcin compared to the control group. The alendronate group also showed significant increases in the total body, total hip, lumbar spine and femoral neck BMDs at the end of study compared to the control group. In the curcumin + alendronate group, BALP and CTx levels decreased and osteocalcin levels increased significantly at the end of study compared to the control and alendronate groups. BMD indexes also increased in four areas significantly at the end of study compared to the control and alendronate groups.

**Conclusion:**

The combination of curcumin and alendronate has beneficial effects on BMD and bone turnover markers among postmenopausal women with osteoporosis. Arch Endocrinol Metab. 2018;62(4):438-45

## INTRODUCTION

One of the main health problems for postmenopausal women is a progressive loss of bone density and, possibly, osteoporosis (1). Studies have shown that half of these patients suffer from osteoporosis and its associated complications, such as bone fractures. Studies have demonstrated that most changes of bone density in postmenopausal women are due to the hormonal changes associated with ovarian function after menopause (2). Despite a growing trend of osteoporosis in postmenopausal women, effective therapeutic and preventive measures have yet to be developed.

Bisphosphonates and hormone replacement therapy (HRT) are two main methods to prevent or treat osteoporotic women's postmenopausal problems. The use of HRT is limited due to its cardiovascular complications, increased risk of breast cancer and vaginal bleeding (3). Long-term bisphosphonates application raises concerns about microdamage accumulation and excessive acceleration of mineralization, osteonecrosis of the jaw and atypical insufficiency fractures in the skeleton system (4). These disadvantages explain why so many studies focus on finding new anti-osteoporotic treatments.

Natural substances can be complementary or alternative means to prevent and treat osteoporosis (5). Curcumin, derived from the turmeric plant *(Curcumin longa* L.), has been shown in laboratory studies to have anti-inflammatory, antioxidant, antifungal and chemopreventive effects (6). Recently, the effects of curcumin on bone remolding and metabolism have been reported in animal studies (7). The anti-osteoporotic effects of curcumin include inhibiting osteoclastogenesis by activating NF-kB ligand (8), inhibiting osteoclasts proliferation, and increasing osteoclasts apoptosis (9). Curcumin's protective effect against the bone deteriorations, induced by glucocorticoids through the activation of microRNA-365 via the suppression of matrix metalloproteinase-9 (MMP-9), has also been reported (10). Studies have also reported that curcumin produces beneficial changes in bone turnover and bone strength (11).

Despite the beneficial effects of curcumin on bone metabolism, the anti-osteoporotic effects of curcumin have not been studied comprehensively in postmenopausal women with osteoporosis. An experimental study reported that the combination of alendronate and curcumin had beneficial effects on bone remodeling and bone mechanical strength in ovariectomized rats (12). Another study showed significant changes in bone turnover and bone strength in a rat model of postmenopausal osteoporosis reported for curcumin (13). Randomized clinical trial (RCT) study reported that treatment of postmenopausal women with alendronate suppressed bone turnover and increased the BMD (14,15). Also, alendronate can reduce the incidence of vertebral fractures in these patients (16). The safety and efficacy have been reported in long term (10-year) treatment with alendronate in RCTs (17). Despite the inhibitory effects of curcumin on the osteoporotic process in various conditions, the effects of curcumin have not been investigated in the treatment or prevention of osteoporosis in postmenopausal women. The current study investigated the combined effects of curcumin and alendronate on bone turnover markers and bone densitometric features related to osteoporosis in postmenopausal women.

## SUBJECTS AND METHODS

### Patient selection and study design

This randomized, double-blind clinical trial study was conducted in an outpatient clinic, specialized in osteoporosis treatment. The study's participants were among healthy postmenopausal women, 55 to 65 years of age, who had at least 5 years since their last menstrual period. In the present study osteoporosis was diagnosed by bone mineral densitometry that was defined by both WHO and applied protocol by previous studies (18). Bone density which was 2.5 SD or more below the young normal mean (T score <-2.5) was classified as showing osteoporosis, while a BMD T score between −1 and −2.5 was classified as showing osteopenia. The WHO definition is applied to postmenopausal women using DXA measurement at the spine, hip or forearm. The aims and scope of the study were orally explained to all subjects, and written informed consent was obtained from all participants. The study was confirmed by the ethics committee of Ilam University of Medical Sciences according to the ethical issues of clinical trials (code no: Ir.medilam.rec.1394.184). The study was registered with the Iranian registry of clinical trials (code no: IRCT2016030424308N1) and the study duration was 12 months. All subjects were identified as new cases of osteoporosis and had not yet received any associated treatment. Exclusion criteria in the current study were as follows: history of bone metabolic diseases; application of drugs affecting bone and metabolism such as bisphosphonates, calcium, and vitamin D, current or past vitamin D deficiency; hormonal problems and HRT; coagulopathy and thromboembolic diseases; cardiovascular and cereberovascular diseases; history of drug abuse, smoking or alcohol use and any unwanted reaction or drug side effect during study. Those who could not continue until the end of study were also excluded. Sixty qualified patients were randomly divided into 3 equal groups of control, alendronate, and alendronate + curcumin (the eligible patients entered into the 3 mentioned groups one by one respectively). The alendronate was administered by 5 mg/day dose, determined from a previously study (15), and the curcumin was administered by 110 mg/day dose (11); both were given for 12 months. All subjects also received calcium supplements (1,000-1,500 mg/day) of calcium carbonate. The control group only received calcium supplements as described above. The drugs dosages applied in this study were the same as used by previous studies in which the effectiveness and safety of the drugs were confirmed. Patients were advised to report any unwanted reaction or drug side effects promptly and in case of need refer to our clinic or emergency department. One of the excluding criteria for participants was any unwanted reaction or drug side effect during drug application. All participants completed the study period without dropping out.

### BMD measurement

BMDs of the lumbar spine (L1-L4), femoral neck, total hip and total body were determined by dualenergy X-ray absorptiometry (DXA) at the beginning of the study and after 6 months using equipment manufactured by a single supplier (Hologic, Waltham, MA). The raw BMD data from all subjects were pooled for analyses. The study defined osteoporosis according to World Health Organization (WHO) criteria (19).

### Bone turnover markers assay

To evaluate the biomarkers, fasting blood samples were obtained from all subjects at the beginning and end of study. The obtained blood samples were centrifuged (1,000 rpm for 10 min) and stored -at −70°C to measure the biomarkers. All assessments were performed in a central laboratory. For the bone formation assay, this study measured the level of BALP (Hybritech, San Diego, CA) and osteocalcin (CIS Biointernational, France) using the radioimmunoassay systems method (18). For the bone resorption marker, this study analyzed the level of urine C-terminal cross-linking telopeptide of type I collagen (Crosslaps, Osteometer A/S and Copenhagen, Denmark) (18).

### Statistical analysis

The quantitative and qualitative data are presented as the mean ± standard deviation (SD) and frequency, respectively. T test, Chi-square and ANOVA were applied for comparison of the results appropriately. Statistical analysis of data was performed by SPSS 19 (SPSS Inc, Chicago, USA) software. A *p* value < 0.05 was considered significant.

## RESULTS

### Patients’ data

The mean ± SD years of postmenopausal state in patients treated with alendronate (10.40 ± 1.50 years) and alendronate + curcumin (9.85 ± 1.89 years) did not statistically differ from the control group (10.45 ± 1.90 years, *p* = 0.669). The mean ± SD of estradiol levels among alendronate (23.64 ± 10.61) and alendronate + curcumin (26.53 ± 9.11) groups also did not show any significant difference to the control group (25.63 ± 9.54, *p* = 0.531). The mean ± SD of the follicle stimulating hormone (FSH) levels were not significantly different between groups (*p* = 0.874). The levels of markers related to bone metabolism and osteoporosis (such as calcium, ALP and phosphorus) also did not differ significantly among the three groups (*p* = 0.459, *p* = 0.621 and *p* = 0.341) (Table 1).

**Table 1:** Basic information of participants

Variables	Control	Alendronate	Alendronate+Cur	P value
Age (year)	58.55 ± 12.45	60.50 ± 11.95	58.00 ± 10.78	0.772
BMI (kg/m^2^)	24.95 ± 3.14	24.55 ± 3.60	24.50 ± 5.70	0.840
Postmenopausal duration (year)	10.45 ± 1.90	10.40 ± 1.50	9.85 ± 1.89	0.669
Estradiol (pg/mL)	25.63 ± 9.54	23.64 ± 10.61	26.53 ± 9.11	0.531
FSH (mIU/mL)	87.43 ± 16.43	79.13 ± 21.43	83.54 ± 18.43	0.874
ALP (IU/L)	126.32 ± 42	129.43 ± 37	122.24 ± 27	0.621
Calcium (mg/dL)	9.32 ± 0.34	9.04 ± 0.51	9.21 ± 0.63	0.459
Phosphorus (mg/dL)	4.3 ± 1.3	4.76 ± 0.76	4.7 ± 0.61	0.341
Total number	20	20	20	

BMI: body mass index; FSH: follicle-stimulating hormone.

### Effect of alendronate and curcumin on bone turnover markers

**Figure 1:**
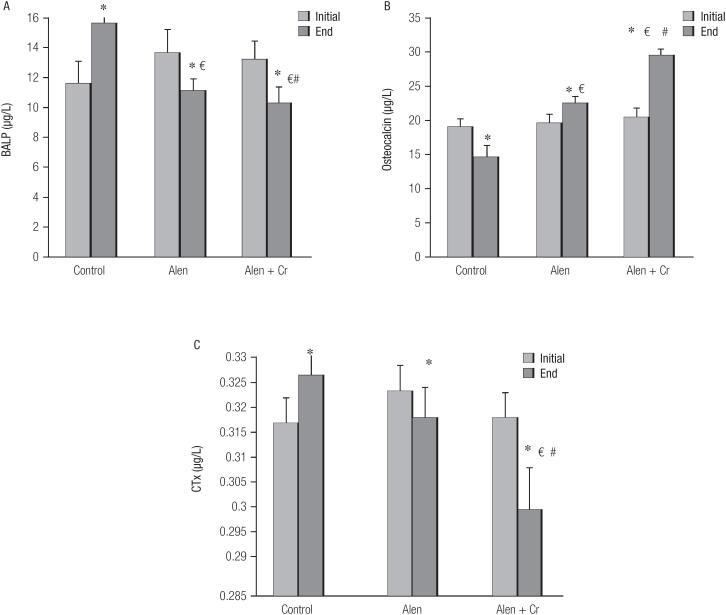
Effect of different drug groups on bone turnover markers in postmenopausal women with osteoporosis. * = p < 0.05: end level compared with initial of study in control group. € = p < 0.05: end level compared with initial of study in alendronate. # = p < 0.05: end level compared with end of study in alendronate + curcumin group. BALP: bone-specific alkaline phosphatase, CTx: C-terminal cross-linking telopeptide of type I collagen.

The mean ± SD of bone turnover markers among differennt groups is shown by Figure 1. Figure 1A shows that by the end of study, the BALP of patients in the control group (13.96 ± 1.1 μg/L, 95% CI: 12.0315.1) slightly increased compared to the baseline (13.29 ± 1.5 μg/L, 95% CI: 12.65-14.3) (*p* = 0.432). However, in both groups of alendronate alone and in combination with curcumin, the BALP level at the end of study decreased compared to the beginning (11.04 ± 0.9 pg/L, 95% CI: 9.02-13.1 vs. 13.54 ± 1.6 pg/L, 95% CI: 11.6-15.1, *p* = 0.023 and 13.16 ± 1.3 pg/L, 95%CI: 10.05-14.6, vs. 10.2 ± 1.2 pg/L, 95%CI: 9.0212.4, *p* = 0.012 respectively). The BALP level of the alendronate group was also significantly lower than the BALP level of the control group by the end of study (*p* = 0.030). Also, the BALP level at the end of study in the alendronate + curcumin group differed significantly from the alendronate only and control groups at the end of study (*p* = 0.020, *p* = 0.035, respectively).

Figure 1B shows the osteocalcin results for the three groups. The mean ± SD of osteocalcin increased at the end of study compared with the baseline in the control group, although this increase was not statistically significant (19.37 ± 1.7 pg/L, 95%CI: 19.07-20.05 vs. 18.95 ± 1.3 pg/L, 95%CI: 18.87-20.01, *p* = 0.432). In the alendronate group, the level of osteocalcin at the end of study decreased compared to the baseline (19.45 ± 1.2 pg/L, 95%CI: 19.02-21.3 vs. 19.75 ± 1.4 pg/L, 95%CI: 19.12-21.6, *p* = 0.620). Similarly, the mean ± SD of osteocalcin in the alendronate + curcumin group level at the end of study decreased compared to baseline. This decrease was not statistically significant (19.02 ± 1.1 pg/L, 95%CI: 17.06-22.14 vs. 20.3 ± 1.3 pg/L, 95%CI: 18.4-23.1, *p* = 0.231).

CTx changes are shown in Figure 1C. At the end of study, the levels of CTx marker in the control group significantly increased compared to the baseline (0.326 ± 0.027 pg/L, 95%CI: 0.12-0.48 vs. 0.317 ± 0.033 pg/L, 95%CI: 0.22-0.53, *p* = 0.020). Unlike the control group, the CTx levels in the alendronate group, at the end of study showed a significant reduction compared to the baseline (0.318 ± 0.012 pg/L, 95%CI: 0.19-0.44 vs. 0.323 ± 0.011 pg/L, 95%CI: 0.25-0.46, *p* = 0.043). Levels of CTx in alendronate + curcumin group showed a significant reduction at the end of study compared to the baseline (0.301 ± 0.017 pg/L, 95%CI: 0.27-0.42 vs. 0.318 ± 0.033 pg/L, 95%CI: 0.23-0.35, *p* = 0.013). The mean serum level of CTx in the alendronate + curcumin group was also significantly less than its level in the alendronate alone and control groups (*p* = 0.020, *p* = 0.037, respectively).

### Effect of alendronate and curcumin on bone densitometry

**Figure 2:**
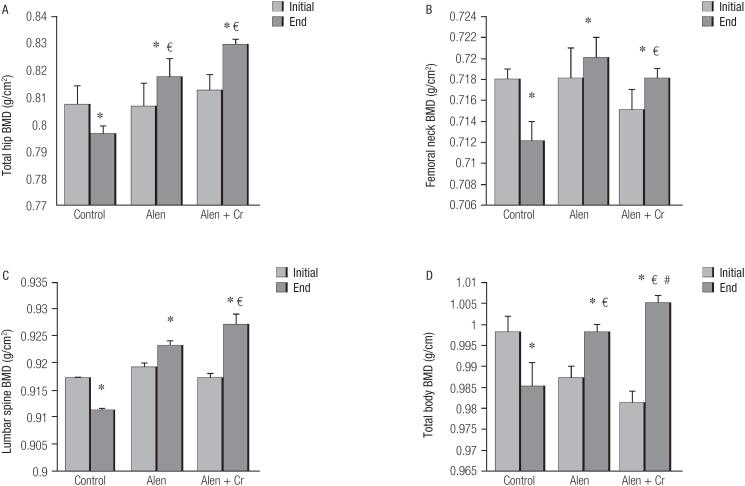
Effect of different drug groups on bone mineral density (BMD) in postmenopausal women with osteoporosis. * = p < 0.05: end level compared with initial of study in control group. € = p < 0.05: end level compared with initial of study in alendronate. # = p < 0.05: end level compared with end of study in alendronate + curcumin group.

Densitometric changes of the total hip BMD are shown by Figure 2A. The mean ± SD of BMD decreased significantly at the end of study compared to the baseline (0.796 ± 0.003, 95%CI: 0.74-0.82 vs. 0.807 ± 0.007, 95%CI: 0.78-0.83, *p* = 0.031). In the alendronate alone and alendronate + curcumin groups, the mean ± SD of BMD showed a significant increase at the end of study compared to the baseline (0.817 ± 0.007, 95%CI: 0.77-0.84 vs. 0.806 ± 0.009, 95%CI: 0.76-0.83, *p* = 0.001 and 0.829 ± 0.002, 95%CI: 0.76-0.84 vs. 0.812 ± 0.006, 95%CI: 0.75-0.83, *p* = 0.01, respectively). Changes in the total hip BMD (Figure 2B) in the alendronate alone and alendronate + curcumin groups significantly increased compared to the control group at the end of study *(p* = 0.035, *p* = 0.046, respectively). The total hip BMD level in the alendronate + curcumin group showed a significant increase compared to the BMD level in the alendronate group (*p* = 0.044).

Changes in the femoral neck BMD (Figure 2C) were almost similar to the total hip BMD results; the mean ± SD of the BMD in the control group was significantly lower at the end of study than the baseline (0.712 ± 0.002, 95%CI: 0.69-0.74 vs. 0.718 ± 0.008, 95%CI: 0.70-0.73, *p* = 0.024). In the alendronate group, the mean ± SD of the femoral neck BMD at the end of study also increased compared to the baseline (0.720 ± 0.002, 95%CI: 0.66-0.78 vs. 0.718 ± 0.003, 95%CI: 0.67-0.79, *p* = 0.56). The alendronate + curcumin group also showed increased levels in the femoral neck BMD at the end of study compared to the baseline (0.718 ± 0.001, 95%CI: 0.67-0.78 vs. 0.715 ± 0.002, 95%CI: 0.63-0.75, *p* = 0.063). Changes in the BMD in both alendronate and alendronate + curcumin groups compared to the control group were statistically significant (*p* = 0.025, *p* = 0.030, respectively).

Changes in the total body BMD are shown in Figure 2D. Like the other variables, the mean ± SD values of BMD in the control group decreased significantly at the end of study compared to the baseline (0.985 ± 0.006, 95%CI: 0.91-1.2 vs. 0.998 ± 0.004, 95%CI: 0.95-1.3, *p* = 0.024). The mean ± SD values of total body BMD among alendronate and alendronate + curcumin groups increased significantly at the end of study compared to the baseline (0.998 ± 0.002, 95%CI: 0.97-1.34 vs. 0.987 ± 0.003, 95%CI: 0.94-1.42, *p* = 0.022 and 1.005 ± 0.002, 95%CI: 0.98-1.45 vs. 0.981 ± 0.003, 95%CI: 0.93-1.33, *p* = 0.020). The mean ± SD values of BMD in the alendronate and alendronate + curcumin groups also significantly increased compared to the control group (*p* = 0.034 and *p* = 0.014, respectively).

## DISCUSSION

Postmenopausal women are commonly at risk of a progressive loss of bone density and, possibly, osteoporosis. The aim of this study was to evaluate the combined effects of curcumin and alendronate on BMD and bone turnover markers in postmenopausal women with osteoporosis. Studies have shown that aging is associated with reduced BMD and bone tissue microarchitecture destruction and that these changes increase bone fragility (20). These destructive and progressive changes occur more rapidly in postmenopausal women. Postmenopausal women, due to hormonal deficiencies, fail to achieve peak bone mass or bone mass is more readily destroyed (21). The results of this study showed that markers related to bone turnover (such as BALP and CTx) increased significantly among postmenopausal women in the control group at the end of study compared to other groups, while osteocalcin decreased in this group. Similarly, the densitometric results showed that BMD indices in postmenopausal women at the end of study significantly decreased compared to other groups. The mechanism driving these changes indicated various factors and showed that estrogen deficiency in postmenopausal women leads to decreased BMD through the thinning of bone trabeculae. Thus, when estrogen decreased, differentiation and proliferation of osteoclasts dramatically increased (22). According to this mechanism, one means of preventing osteoporosis in postmenopausal women is HRT and in this field, bisphosphonates are the most important and well-known drugs. This study's results showed that alendronate effectively reduced the levels of BALP and CTx and increased osteocalcin by the end of study. When compared to the values at the study's onset, these changes were significantly different for the BALP and osteocalcin markers but were not significantly different for the CTx. The results showed that the mean level of BALP and CTx in the alendronate group decreased significantly after one year. The osteocalcin levels in the alendronate group also increased significantly compared to the control group. These results mean that alendronate could effectively decrease bone turnover markers in postmenopausal women. A densitometric study also showed that the alendronate group exhibited a significantly increase in total body, waist, hip and lumbar spine BMDs at the end of study compared to the onset. This outcome revealed that alendronate is beneficial to preventing the development of osteoporosis in postmenopausal women. Previous clinical and laboratory studies support this study's finding. For example, a study by Iwamoto et al demonstrated that treating women, with and without type 2 diabetes, with alendronate reduced the level of alkaline phosphatase (ALP) and increased lumbar spine BMD compared to control group (23). That study also examined the possible side effects of alendronate and did not observe any instances of complications such as atrial fibrillation, atypical diaphyseal femoral fracture or jaw osteonecrosis. Another study by Iwamoto showed that alendronate significantly decreased the average levels of ALP and NTX and increased lumbar spine BMD in postmenopausal women after one year. That study also showed that alendronate reduced the incidence of vertebral and non-vertebral fractures (24). Correspondingly, the present study's results showed that treatment with alendronate for one year reduced bone turnover markers and increased the BMD values of the total body, waist, hip, and lumbar spine. Experimental investigations have reported evidences supporting the assertion that alendronate inhibits osteoporosis. Lindtner and cols. examined the osteoanabolic effects of alendronate and zoledronate on the process of human bone marrow stromal cells (hBMSCs) osteogenesis in women with osteoporosis and that these compounds could increase the differentiation of hBMSC classes. That study also reported that these compounds could reduce the osteopontin marker levels in hBMSCs lines (25). While most studies on the effects of alendronate on osteoporosis in postmenopausal women utilized monotherapy, Hejdova and cols. examined the beneficial effects of combining alendronate and calcitonin on BMD in postmenopausal women (26). Although several studies have reported alendronate's antiresorptive properties and it has been used as a first-line treatment for osteoporosis, possible side effects have raised concerns about the benefits and harms of bisphosphonates. In fact, studies have shown that long-term use of BPs was associated with an increased risk of stroke and deep vein thrombosis (DVT); this effect is probably caused by an estrogen-induced effect due to release of clotting factors (27–29). As for these concerns the current study aimed to combine alendronate with curcumin, which has reportedly shown beneficial effects on osteoporosis in clinical and *in vitro* investigations and can inhibit alendronate's side effects through its antioxidant properties (30). For example, studies have shown that curcumin can inhibit the activity of the tissue factor involved in thrombotic disorders and decrease platelet activity or inhibit platelet aggregation (31). In addition to curcumin's advantage in reducing the side effects of BPs, several studies have reported curcumin's beneficial effects in preventing osteoporosis in experimental and clinical models (32). The present study's results clearly showed the efficacy of curcumin in combination with alendronate on bone turnover markers and densitometric indices. The results showed that curcumin in combination with alendronate significantly decreased BALP and sCTx markers at the end of study compared to the control group. The present study also found that curcumin in combination with alendronate significantly increased BMD compared to the alendronate and control groups alone. This study's results are the first to report the beneficial effects of a therapy combining curcumin with a BP in postmenopausal women with osteoporosis. To date, no study had been conducted with this type of combination therapy. Most studies had been carried out as monotherapy and administered curcumin or alendronate separately. Similar results were observed in Cho and cols.'s study, which showed that eight weeks application of high doses of curcumin decreased the ALP, telopeptide-C, and osteocalcin markers in ovariectomized rats compared to the control group. That study also showed that curcumin could reveal a dose-dependent increase in BMD compared with the control group (33). Another study by Cho and cols. showed that curcumin in combination with alendronate reduced the bone turnover markers (ALP and CTX) compared with a control group in ovariectomized rats. In another study by Kim and cols, models of ovariectomized rats demonstrated that curcumin inhibited osteoclastogenesis by increasing the activity of a receptor-mediated of NF-kB (34). While previous clinical trial studies have reported some beneficial effects of curcumin on other musculoskeletal problems such as osteoarthritis, cartilage disease (Cartilaginous), and sarcoma pathology of skeletal muscle (35), our study is the first to report the effects of a combination therapy on osteoporosis among postmenopausal women. The exact mechanisms through which curcumin significantly inhibits osteoporosis have not been determined, but evidences suggest that curcumin inhibits the process of osteoporosis by a series of multi-stage activities. The stages of this process include inhibiting NF-kB and RANKL signaling, inhibiting the production of nitric oxide and reactive oxygen species (ROS) and synthesizing inflammatory cytokines. Curcumin also may inhibit the differentiation and proliferation of osteoclasts (5). This study revealed that both curcumin and alendronate improved the densitometric status and bone remodeling markers of postmenopausal women; however, these findings were significantly higher among those who received curcumin and alendronate as combined compared to the groups receiving these medications lonely. This result indicated that combined application of curcumin and alendronate may be a useful option for prevention and treatment of osteoporosis among postmenopausal women. This study had some limitations such as: groups treated with curcumin alone and the effects of curcumin were not revealed because of limited screening and patient numbers as well as patients’ incompatibility. The study also did not examine the fracture risk of women in the study and only examined bone turnover markers and BMD. It is hoped that these points will be examined in future clinical trial studies.
